# Heterogeneous Salmonella typhi transmission within a household: genomic insights from a chronic carrier

**DOI:** 10.1099/jmm.0.002070

**Published:** 2025-09-22

**Authors:** Rushana Hussain, George Stenhouse, Amina Ismail Ahmed, Timothy J. Dallman, Satheesh Nair, Matthew Bird, Duncan Berger, Gherard Batisti Biffignandi, Kate S. Baker, Gauri Godbole, Marie Anne Chattaway

**Affiliations:** 1 Microbiology Department, Royal Bolton Hospital Foundation Trust, Bolton, UK; 2 Gastrointestinal Bacteria Reference Laboratory, United Kingdom Health Security Agency, London, UK; 3 Gastrointestinal Infection Health Protection Research Unit, University of Liverpool, Liverpool, UK; 4 Faculty of Veterinary Medicine, Institute for Risk Assessment Sciences (IRAS), Utrecht University, Utrecht, Netherlands; 5 Healthcare Associated Infections and Antimicrobial Resistance Health Protection Research Unit, University of Oxford, Oxford, UK; 6 Genomic and Enabling Data Health Protection Research Unit, University of Warwick, Coventry, UK; 7 Department of Genetics, University of Cambridge, Cambridge, UK; 8 Gastrointestinal Pathogens and Food Safety (One Health), United Kingdom Health Security Agency, London, UK

**Keywords:** carriage, evolution, genomics, public health, resistance, Typhi

## Abstract

**Introduction.** Household outbreaks of *Salmonella enterica* serovar Typhi (*S.* Typhi) typically involve genetically similar strains, often within a 0–5 single-nucleotide polymorphism (SNP) single linkage cluster. However, unusual genetic heterogeneity may indicate more complex transmission dynamics. This case represents an instance of household transmission facilitated by an asymptomatic carrier harbouring genetically diverse *S.* Typhi strains. A healthy adult developed severe typhoid requiring hospitalization, and two children required treatment. Public health epidemiological connections among the cases supported the findings of the phylogenetic analysis.

**Hypothesis/Gap Statement.** We hypothesized that a household cluster of *S.* Typhi infections, displaying greater-than-expected genetic diversity, may have originated from a chronic carrier with a diverse in-host bacterial population – an under-recognized transmission route.

**Aim.** To investigate the source and genomic diversity of a household cluster of *S.* Typhi cases with no recent travel history and to assess the role of asymptomatic carriage in transmission.

**Methodology.** We conducted detailed contact tracing, epidemiological investigations and whole-genome sequencing on isolates from four household cases. An asymptomatic contact, with a history of *S. Typhi* infection and recent travel to Pakistan, underwent enhanced sampling and genomic analysis of multiple *S.* Typhi isolates.

**Results.** The four household cases formed a 25-SNP single linkage cluster, inconsistent with typical isogenic clustering. Genomic analysis of multiple isolates from the asymptomatic carrier revealed a genetically diverse *S.* Typhi population with evidence of in-host evolution. Two case isolates were nested within the genomic diversity of the carrier’s isolates. Epidemiological investigations identified no alternative sources of infection.

**Conclusion.** This case series highlights the complexity of defining *S.* Typhi transmission using discrete SNP thresholds. While epidemiological links suggested a single source, phylogenetic analysis revealed notable genetic diversity among the strains. The findings underscore the public health risks posed by chronic carriers harbouring diverse *S.* Typhi populations and the complications associated with typhoid transmission and disease severity.

**Impact Statement**

In this article, we demonstrate the use of whole-genome sequencing to establish *Salmonella* Typhi acquisition from a UK chronic carrier in a family cluster. Using epidemiological and genomic analysis, we were able to recommend that the use of single-nucleotide polymorphism- or allele-based clustering methods as part of case definition may not always be appropriate, especially in heterogeneous outbreaks or clusters. Sequencing of the *S*. Typhi strains in this family cluster produced a heterogeneous phylogeny showing multiple microbiological sub-clusters that would not normally have indicated transmission. The clinical, epidemiological investigation alongside the detection of multiple sub-clusters of *S*. Typhi shows that the chronic carrier was likely to have been infected a number of years previously. Contamination from an endemic source with a mixed population of strains would be unusual in an *S*. Typhi carrier, and further genomic analysis provided evidence for in-host evolution of the organism. We, therefore, advocate for the integration of genomic typing methods with epidemiological investigation, cautioning against reliance solely on genetic relationships for contact tracing in outbreaks. Further research aimed at enhancing the detection of *S*. Typhi chronic carriers is warranted to elucidate potential transmission sources and improve outbreak management strategies.

## Data Summary

FASTQ sequences were deposited in the National Center for Biotechnology Information (NCBI) Short Read Archive under the BioProject PRJNA248792 (https://www.ncbi.nlm.nih.gov/bioproject/?term=248792). Refer to Table 1 and Table S1 for SRR accession numbers.

**Table 1.** Genetic variation of resistance markers of *S.* Typhi isolates from the cases and chronic carriers The reference number is based on the patient (A–D) and the individual colony number that was isolated from the original clinical sample and sequenced.

Patient	Reference no.	Case/carrier	Sex	Age	SRA	Sample date	Plasmid replicon	SNP address	*β*-Lactamases	Aminoglycoside	Fluoroquinolone	Trimethoprim	Sulphonamide	Chloramphenicol
A	A1	Case	F	20	SRR12672341	05/09/2020	IncQ1	1.1.1.1.58.784.1392	blaTEM-1	aac(6)-Iy;aph(6)-Id,strA;strB	gyrA_[83:S-F];ParC_[78:G-D]	dfrA-7	sul-1; sul-2	catA-1
B	B1	Case	F	15	SRR12806902	23/09/2020	n/a	1.1.1.1.58.787.1397	–	aac(6)-Iy	gyrA_[83:S-F]	–	–	–
C	C1	Case	M	11	SRR12743431	11/09/2020	IncQ1	1.1.1.1.58.785.1394	blaTEM-1	aac(6)-Iy;aph(6)-Id,strA;strB	gyrA_[83:S-F]	dfrA-7	sul-1; sul-2	catA-1
D	D1-1	Carrier	F	78	SRR12807764	24/09/2020	IncQ1	1.1.1.1.58.788.1398	blaTEM-1	aac(6)-Iy;aph(6)-Id,strA;strB	gyrA_[83:S-F]	dfrA-7	sul-1; sul-2	catA-1
D	D2-1	Carrier	F	78	SRR13163225	11/11/2020	IncQ1	1.1.1.1.58.787.1406	blaTEM-1	aac(6)-Iy;aph(6)-Id,strA;strB	gyrA_[83:S-F]	dfrA-7	sul-1; sul-2	catA-1
D	D3-1	Carrier	F	78	SRR13833611	02/02/2021	IncQ1	1.1.1.1.58.787.1434	blaTEM-1	aac(6)-Iy;aph(6)-Id,strA;strB	gyrA_[83:S-F]	dfrA-7	sul-1; sul-2	catA-1
D	D3-2	Carrier	F	78	SRR15334932	02/02/2021	IncQ1	1.1.1.1.481.801.1429	blaTEM-1	aac(6)-Iy;aph(6)-Id,strA;strB	gyrA_[83:S-F];ParC_[78:G-D]	dfrA-7	sul-1; sul-2	catA-1
D	D3-3	Carrier	F	78	SRR13772107	02/02/2021	n/a	1.1.1.1.480.798.1424	–	aac(6)-Iy	gyrA_[83:S-F];ParC_[78:G-D]	–	–	–
D	D3-4	Carrier	F	78	SRR13833659	02/02/2021	IncQ1	1.1.1.1.58.787.1434	blaTEM-1	aac(6)-Iy;aph(6)-Id,strA;strB	gyrA_[83:S-F]	dfrA-7	sul-1; sul-2	catA-1
D	D3-5	Carrier	F	78	SRR15334714	02/02/2021	IncQ1	1.1.1.1.58.787.1426	blaTEM-1	aac(6)-Iy;aph(6)-Id,strA;strB	gyrA_[83:S-F]	dfrA-7	sul-1; sul-2	catA-1
D	D3-6	Carrier	F	78	SRR15334737	02/02/2021	IncQ1	1.1.1.1.58.799.1425	blaTEM-1	aac(6)-Iy;aph(6)-Id,strA;strB	gyrA_[83:S-F];ParC_[78:G-D]	dfrA-7	sul-1; sul-2	catA-1
D	D3-7	Carrier	F	78	SRR15334694	02/02/2021	IncQ1	1.1.1.1.58.787.1428	blaTEM-1	aac(6)-Iy;aph(6)-Id,strA;strB	gyrA_[83:S-F]	dfrA-7	sul-1; sul-2	catA-1
D	D3-8	Carrier	F	78	SRR13833654	02/02/2021	IncQ1	1.1.1.1.58.787.1434	blaTEM-1	aac(6)-Iy;aph(6)-Id,strA;strB	gyrA_[83:S-F]	dfrA-7	sul-1; sul-2	catA-1
D	D3-9	Carrier	F	78	SRR15340843	02/02/2021	IncQ1	1.1.1.1.58.787.1433	blaTEM-1	aac(6)-Iy;aph(6)-Id,strA;strB	gyrA_[83:S-F]	dfrA-7	sul-1; sul-2	catA-1
D	D3-10	Carrier	F	78	SRR15334775	02/02/2021	IncQ1	1.1.1.1.58.800.1427	blaTEM-1	aac(6)-Iy;aph(6)-Id,strA;strB	gyrA_[83:S-F];ParC_[78:G-D]	dfrA-7	sul-1; sul-2	catA-1
D	D3-11	Carrier	F	78	SRR15334815	02/02/2021	IncQ1	1.1.1.1.483.803.1432	blaTEM-1	aac(6)-Iy;aph(6)-Id,strA;strB	gyrA_[83:S-F]	dfrA-7	sul-1; sul-2	catA-1

Isolate D3-1 was the first isolate after fosfomycin treatment and despite the development of resistance, no additional genetic antibiotic resistance markers were detected.

## Introduction

Typhoid fever is a systemic infection caused by the bacterium *Salmonella enterica* serotype Typhi (*S*. Typhi). It is mainly a disease of the low- and middle-income countries, particularly the Indian sub-continent and Southeast Asia [1]. The organism is transmitted through the faecal–oral route and is associated with poor sanitation and hand hygiene [2].

The incidence of travel-associated typhoid in high-income countries is rising annually, with ~300 cases of enteric fever reported in the UK. The majority (99%) of the cases are associated with recent travel to endemic countries [3]. *Salmonella* Typhi accounts for ~60% of invasive *Salmonella* cases in England, Wales and Northern Ireland, with the majority of cases being associated with travel from Pakistan and India [3].

Typhoidal infections are typically sub-acute with non-specific symptoms, leading to invasive infections associated with bacteraemia. Symptoms can develop over 6 weeks prior to bacteraemia and can relapse 4 weeks post-treatment [4,5]. Mortality is reduced by treating infections with antibiotics and providing supportive rehydration [1].

Most infections are cleared, with faecal shedding occurring in convalescent carriers from 3 weeks to 3 months post-infection and in temporary carriers from between 3 and 12 months [6,9]. However, ~2–5% of typhoid patients fail to fully clear the infection within 1 year of recovery, instead progressing to a state of chronic carriage [10,11]. Bacterial persistence, characterized by chronic and relapsing infections, poses a significant threat to human health, as these infections can lead to family and community outbreaks, increased morbidity and mortality and, in the case of *S*. Typhi, are associated with gallbladder, pancreas or bowel carcinomas [8,12, 13]. Although further studies are needed to understand the molecular mechanisms and host factors that induce the carrier state [14], numerous clinical indicators link persistent *Salmonella* carriage to the human gallbladder [11].

This case represents one of the earliest documented instances of household transmission facilitated by an asymptomatic carrier harbouring genetically diverse strains. Consequently, a healthy adult developed severe typhoid, necessitating hospitalization and two children required treatment. Public health epidemiological connections among cases are presented to support the findings of the phylogenetic analysis. However, while these links suggest a single source for the strains, the phylogenetic analysis reveals significant differences among them. This dichotomy underscores the complexity involved in employing discrete single-nucleotide polymorphism (SNP) thresholds to ascertain related cases of *S*. Typhi. Additionally, this case series underscores the potential public health hazards posed by typhoid carriers and the various complications associated with this illness.

## Methodology

### Epidemiology and cases

The clinical team reviewed a cluster involving four individuals from an extended family, where one individual required hospitalization, two others exhibited symptoms and one was initially asymptomatic, and they were considering it a potential household outbreak. Patient travel and clinical symptoms were obtained from hospital case notes and the UK Health Security Agency’s (UKHSA, formerly known as Public Health England) using an enhanced surveillance questionnaire. The local health protection units routinely follow up on all enteric fever contacts, including screening those within the household.

### Microbiology and typing

Blood and stool culture grew non-lactose fermenting colonies on MacConkey agar, identified as *Salmonella* species by matrix-assisted laser desorption ionization time-of-flight (MALDI-TOF) MS at the Royal Bolton Hospital diagnostic laboratory. * Salmonella* serological reactions with O9, d and Vi antibodies indicated *S*. Typhi.

Presumptive isolates of *S*. Typhi (patients A, B, C and D1-1) from a cluster of four patients were sent to UKHSA Gastrointestinal Bacteria Reference Unit (GBRU) for confirmation and further characterization using a typhoidal PCR [15] and whole-genome sequencing as previously described [6,16, 17]. For case D, there were three samples: the likely carrier index case sample (D1-1 from 24 September 2020), a post-treatment isolate (D2-1 from 11 November 2020) and a symptomatic infection stool sample ~5 months later (D3-1 from 02 February 2021) (Table 1). The symptomatic stool sample, post-fosfomycin treatment, was sent to the GBRU for sequencing investigations of multiple isolates (*n*=11, D3-1 isolates, Table 1), to explore the genetic diversity of *S*. Typhi shedding in case D.

*Salmonella* serovar determination was predicted based on the *Salmonella* eBURST group (eBG) and sequence type (ST) and checked against a validated UKHSA database [18]. SNPs were identified with respect to the *S*. Typhi reference genome Ty2 (AE014613.1) using SnapperDB (v.2.6.0) [18]. SNPs were classified as coding or non-coding, and the impact of the mutation was classified as synonymous, non-synonymous or loss-of-function using SnapperDB. *In silico* identification of genetic antimicrobial resistance (AMR) was conducted for all Illumina reads using GeneFinder (v2.7) (https://github.com/phe-bioinformatics/gene_finder) as previously described [6]. AMRFinderPlus (v3.12.6) was used as a comparative method to confirm the presence and absence of AMR genes [19,20]. PlasmidFinder (v2.1) was used to confirm the presence of plasmid replicons [20] [1]. UKHSA also makes FASTQ sequences publicly available by routinely uploading *Salmonella* sequence data to NCBI BioProject PRJNA248792 (https://www.ncbi.nlm.nih.gov/bioproject/?term=PRJNA248792) and accession numbers are stated in this study (Table 1, Table S1, available in the online Supplementary Material).

Study isolates were also typed according to the previously defined genoTyphi genotyping scheme [21] using Mykrobe (v0.12.1) (https://github.com/Mykrobe-tools/mykrobe), and grouping was visually confirmed on the phylogeny.

Antimicrobial susceptibility testing was performed by the clinical laboratory using the Becton Dickinson (BD) Phoenix system and interpreted using MIC breakpoints according to the European Committee on Antimicrobial Susceptibility Testing (V10) [22].

### Phylogenetic analysis

#### Contextual phylogeny and functional SNP analysis

To assess the relatedness of the *S*. Typhi cluster in context, the evolutionary relationships between *S*. Typhi isolated from the four cases (*n*=16) and 342 reference isolates were selected. Phylogenetic reference isolates were sub-sampled from (1) globally represented *S*. Typhi imported sentinel surveillance strains identified by UKHSA between 2016 and 2019 (*n*=150) [6] and (2) strains from the global *S*. Typhi genotyping scheme (*n*=73) [21] (Table S1) to ensure a full global representation and genotype diversity of *S*. Typhi strains. UKHSA reference isolates were sub-sampled by ST and year to ensure that all sequence types were included and every year was represented proportionally to the ST group size and to cover the full detected diversity of AMR determinants. All *S*. Typhi genotypes were selected for inclusion.

The input core-SNP alignment (10,737 SNPs) was generated by mapping quality-trimmed isolate reads to the complete *S*. Typhi TY2 reference genome (AE014613.1) with bwa mem (v0.7.17-r1188) [23]. Specifically, reads were filtered for quality and duplication, indexed and sorted, with Samtools (v1.15.1) [24,26], duplicate reads marked with Picard (v2.27.4) (https://broadinstitute.github.io/picard/), and clipped with Samclip (v0.4.0) (https://github.com/tseemann/samclip). Quality of read mapping was assessed using Qualimap (v2.2.2-dev) [27] with a minimum required mean read mapping depth of 20. Variant sites of the mapped reads were called using Samtools mpileup, and consensus sequences were defined using bcftools (1.15.1) [26,28]. Bedtools (v2.30.0) was used to mask all known and probable phage regions, defined using the web-based Phaster software [29]. Gubbins (v2.4.1) was used to detect and mask recombination in the alignment [30,31]. The tree was then generated using RAxML-ng v0.6.0 GTR+G evolutionary model [32,33]. A total of 95 SNPs were detected among the *S*. Typhi cluster isolates (*n*=95) and were then analysed for their functional implications.

Since genome degradation has been shown in previous studies to be an important mechanism of evolution in chronic *S*. Typhi infection, indels were identified independently using the same method as the core-SNPs. Functional impact of indels was determined with SnpEff and SnpSift [34,35].

#### Cluster analysis

To perform an accurate detection of epidemiological clusters, a first phylogeny of the 16 study isolates was inferred through the P-DOR pipeline [36]. The quality of all sequence reads was assessed using FastQC (v0.11.9) [37], followed by a two-step trimming process (Trimmomatic v0.39) and seqtk (v1.3-r106) [38]. Following trimming, read quality required the exclusion of all reference isolate unpaired reads from further analysis. As P-DOR requires as input the genomes, short reads of the 16 strains were *de novo* assembled using Shovill (github.com/tseemann/shovill). Although P-DOR allows the query strains to be contextualized with a genomic background retrieved from the BV-BRC database [39], only the 16 isolates were considered. In the following step, P-DOR aligned each query strain to the reference genome (AE014613.1) and the core-SNPs (*n*=169) identified – defined as variable sites flanked by conserved bases in all the genomes in the alignment. Then, the phylogenetic tree was inferred using IQ-TREE, including the ModelFinder selection of the best evolutionary model (K3*P*+ASC). The obtained phylogenetic tree is annotated with resistance genes found with AMRFinderPlus [19], considering only the exact matches. The pairwise distribution of SNP distance between isolates was also visualized.

## Results

### Case history

#### Case A

A 20-year-old female was admitted to casualty after presenting with vomiting, diarrhoea, fever and a 2-week history of feeling generally unwell. She also reported a sore throat and dry cough. The patient had no past medical history. Upon examination, she was febrile and had a clinical assessment early warning score of 5 [40]. Briefly, a clinical assessment early warning score of 5 indicates moderate severity. The score is derived from the Royal College’s National Early Warning Score 2 system, which standardizes the assessment of acute illness severity in the National Health Service [40]. A score of 5 suggests that the patient’s condition warrants attention and monitoring, but it does not indicate severe or life-threatening illness.

Initial laboratory investigations showed raised C-reactive protein with transaminitis. Her Coronavirus disease-19 (COVID-19) swab was negative. She was hospitalized and started on intravenous piperacillin-tazobactam. Blood cultures were flagged as positive, at 24 h, for a Gram-negative bacillus. Direct identification using the Bruker MBT Sepsityper® test was performed, though no identification was given, and so broad-spectrum antibiotic treatment of piperacillin-tazobactam was continued. The following day, the sub-cultured organism was identified as *Salmonella* sp*.* by Bruker MALDI-TOF and as an *S*. Typhi based on serology.

Treatment was changed to treat *S*. Typhi with intravenous ceftriaxone for 2 weeks and risk factors were revisited. The patient was HIV negative and reported no recent travel; she did, however, report other family members, the younger two siblings, being unwell with diarrhoea and fever and the recent return (2 months) of her grandmother from Pakistan. Follow-up of all household contacts was requested by the health protection team.

#### Case B

Eight days after case A first presented, a symptomatic household contact, a 15-year-old sibling, reporting a 1-week history of generally feeling unwell and fever, submitted a stool sample for investigation. A blood culture was requested but not provided by the patient. *S*. Typhi was isolated from the sample. Advice was issued for the child to present to A&E at the hospital for assessment; however, parents were hesitant, and further investigations were not possible at the hospital. Therefore, infection control information was issued, and 7 days of per oral (PO) azithromycin was commenced via the General Practitioner (GP). No recent travel abroad was established.

#### Case C

Eighteen days after case A, an 11-year-old asymptomatic household contact, another sibling, also submitted a stool sample for investigations via their GP. Due to the patient being apyrexial, blood cultures were not indicated. *S*. Typhi was isolated from the sample. As the patient was asymptomatic, no further samples were requested, and no treatment was issued, though infection control information was provided. Again, no recent travel abroad was established.

#### Case D

Twenty days after case A presented, *S*. Typhi was isolated from a stool sample requested from an asymptomatic significant contact; case D, the grandmother to cases A, B and C. It was revealed that she would often cook and stay in the same household in these cases. Public health investigations established she had returned from an extended holiday in Pakistan in June 2020, 6 weeks prior to the presentation of case A. She reported no history of illness during or upon return from Pakistan. However, she reported having been diagnosed with and treated for clinically diagnosed typhoid fever on three occasions in Pakistan, once as a child and on two separate occasions over 10 years earlier. Given her previous clinical history, the isolation of *S*. Typhi and travel to an endemic region, she was treated with 7 days of PO azithromycin followed by a PO fosfomycin 3 g sachet once per week for 4 to 6 weeks to aid with eradication of the organism. Fosfomycin was selected for eradication therapy as other options either showed antimicrobial resistance, were intravenous options or were agents associated with antibiotic-associated diarrhoea and *Clostridioides difficile*. Previous studies have supported the use of fosfomycin as a possible treatment option [41], and due to the patient being asymptomatic, combination therapy including fosfomycin was used to attempt eradication.

The patient remained asymptomatic during eradication therapy and upon completion of antibiotic therapy, a repeat stool sample was requested for clearance from which *S*. Typhi was isolated from the clearance stool. However, the antibiogram of the isolate differed from that of the initial isolate, notably in the susceptibility to fosfomycin. The first and second *S*. Typhi isolates (D1-1 and D2-1) were susceptible to fosfomycin, whereas the isolates post-treatment were resistant (D3-1), though no specific resistance mechanism could be established (Table 1, Table S2). The patient was referred for assessment and further investigations to the local infectious disease department; however, she did not attend the follow-up appointment. The infectious diseases team reviewed the patient, and no further investigations were instigated as she remained well and posed a low risk as a contact if she continued to follow infection control measures.

Five months after the initial diagnosis, case D presented at the GP with diarrhoea. A stool sample was obtained from which multiple colony picks of *S*. Typhi were isolated and sequenced (D3-1 to D3-11, Table 1). The patient was treated with 7 days of PO azithromycin.

Public health investigations and questionnaires confirmed person-to-person spread between all four cases in the community, with case D being the source.

### Antibiograms

MIC of isolates from patients A, B and C and the first isolate from patient D (D1-1) initially all had the same MIC pattern, with the exception of trimethoprim for the isolate from patient B (B1, MIC <1 mg l^−1^). The third isolate from patient (D3-1) also had a similar antibiogram to the other isolates except for fosfomycin. This isolate had an MIC of 64 mg l^−1^, this being significantly higher than isolates from the other patients, as well as the first and second isolates from the same patient D1-1 (MIC <16 mg l^−1^) (Table S2). It was suspected that the organism had acquired a mechanism that induced resistance. However, genomic screening of antimicrobial resistant determinants showed no genetically encoded fosfomycin resistance mechanism (Table S2). This would indicate an alternative mechanism of resistance, such as modifications of membrane transporters that prevent fosfomycin from entering the bacterial cell, novel *MurA* mutations or acquisition of plasmid-encoded genes that inactivate fosfomycin not present in current databases. These alterations would not be detected using the genome sequencing undertaken [42].

### Genotyping and genetic diversity

**Fig. 1.**
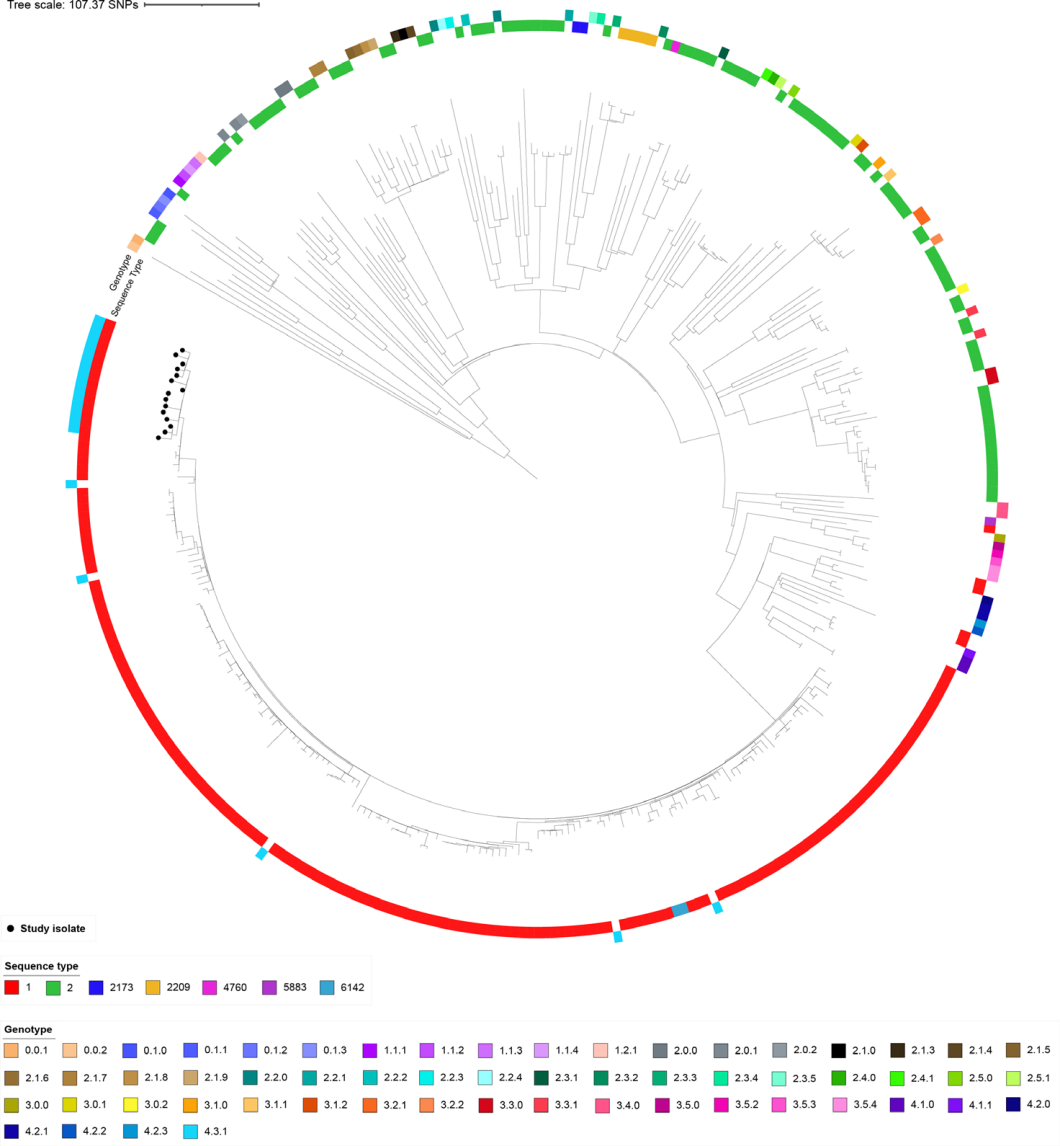
Maximum likelihood tree of *S*. Typhi study isolates among reference isolates from across the known population. The study isolates (black terminal node circles) form a cluster within the sequence type 1/4.30.1 genotype lineage. Reference isolates (no terminal node circle) were sourced from two data sources and originally typed according to two different nomenclatures. UKHAS-identified reference isolates were sequence typed (inner ring), while isolates from Wong *et al*. [21] *S*. Typhi genotyping paper were genotyped (outer ring). Our study isolates were typed according to both schemes and thus have both an ST and genotype.

Isolates from the three cases, A, B and C, and the carrier, case D, were identified by genomic sequencing as *S*. Typhi, ST1, eBG13, genotype 4.3.1.1 (lineage H58), a known multidrug-resistant lineage (Table 1, Fig. 1) [6,43] (Table 1). The close relationship and monophyletic nature of isolates derived from the case cluster were confirmed by a phylogenetic analysis, which placed the cluster isolates in context with both genotypic references (from a defined global population structure) and epidemiological references (subsampling of *S*. Typhi global sentinel surveillance in the UK, Table S1) (Fig. 1).

Despite being part of a monophyletic cluster in the context of global and circulating populations, there was genetic diversity among the isolates from the case cluster. Specifically, based on the determination of isolate SNP addresses (see methods, Table 1), isolates from the original case investigation and treatment period (September 2020 to November 2020) were within only the same 10-SNP cluster (1.1.1.58 .x), not the typical 5-SNP cluster threshold expected to indicate a high likelihood of epidemiological transmission [6,44, 45]. This surprising diversity increased further with the subsequent multi-isolate sampling of case D in February 2022 (D3-1 through D3-11), with four different 10-SNP clusters being represented and isolates only being identical to the 25-SNP threshold level (Table 1).

**Fig. 2.**
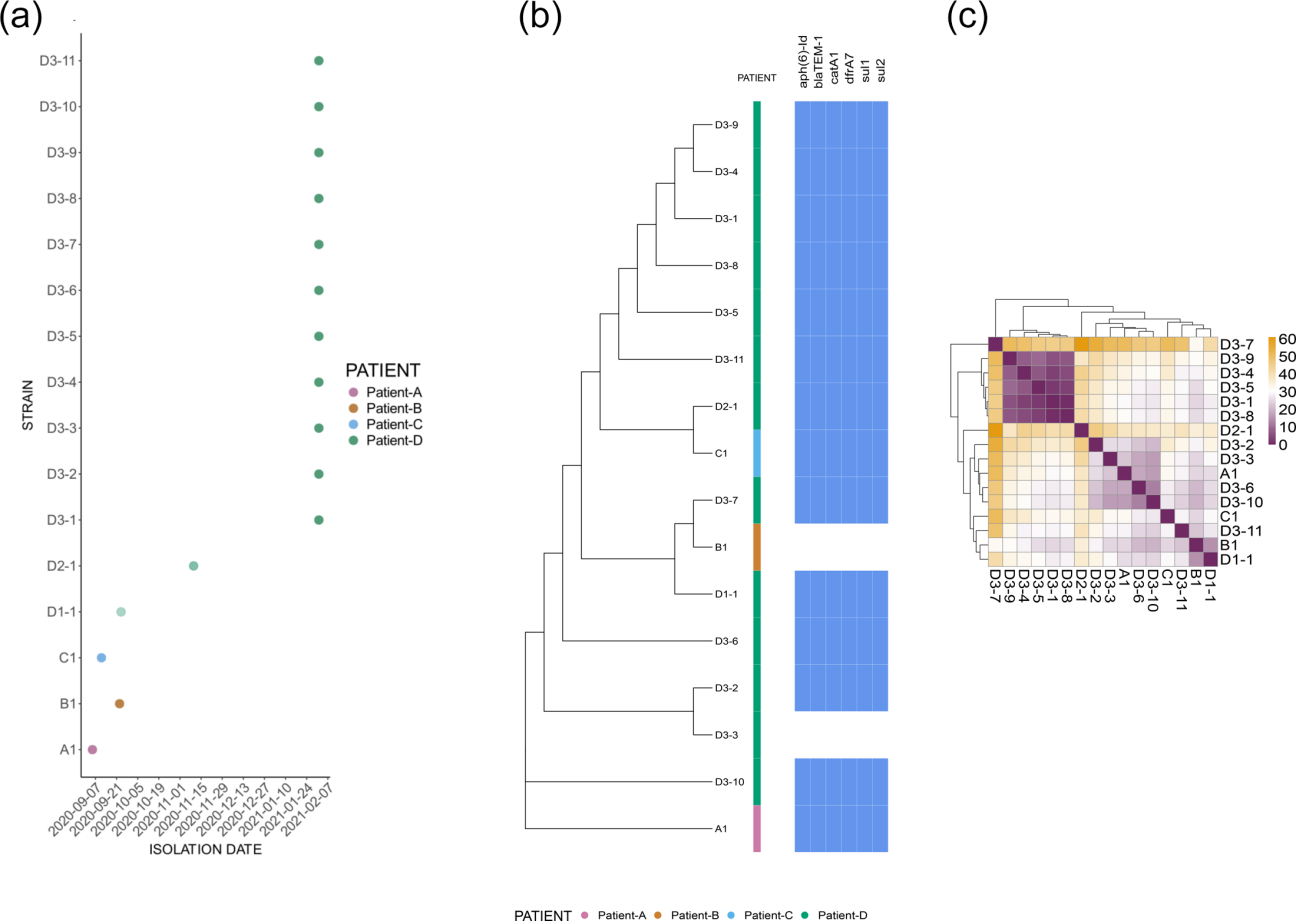
**(a**) Patient-isolate timeline depicting the route of infection transmission. Patient A, pink; patient B, orange; patient C, blue; patient D, green. Multiple colony picks of *S*. Typhi (D3-1 to D3-11) were isolated from patient D on 02 February 2021. (b) Maximum likelihood phylogenetic tree with AMR genotypic profiles. Acquired antimicrobial resistance genotypes detected with exact match are annotated on the tree, showing the presence of *β*-lactams (*bla*TEM-1), aminoglycosides (*aph(6)-Id*), trimethoprim (*dfrA-7*), sulphonamides (*sul1*, *sul2*) and chloramphenicol (*catA-1*). Isolate D3-1 was the first isolate after fosfomycin treatment, and despite the development of resistance, no additional genetic antibiotic resistance markers were detected. (**c**) core-SNP pairwise distance heatmap. Genomic diversity among isolates was larger in case D (range 1–61), compared to cases A, B and C (range 25–32).

To explore the genetic diversity among case cluster isolates in greater detail, another phylogenetic tree was generated to specifically examine the relationships among the 16-case cluster isolates; 1 from each of cases A, B and C and the 13 from case D (Table 1). This revealed a total of 95 SNPs (with respect to the reference) among the isolates and that the diversity among isolates from case D (range 1–61 pairwise SNPs) was larger than the diversity among cases A, B and C (range 25–32 pairwise SNPs, Fig. 2). The variation seen across case D isolates and the nesting of case B-1 and C-1 strains indicates case D as a potential source of infection for these cases, and deeper sampling of case D may have shown additional variation that might have encompassed case A-1. Thus, the epidemiological context, evolutionary relationships and comparable genetic diversity within case D and across all other cases are consistent with case D being a source of infection for the other cases.

We identified eight additional indels in coding regions, present in all study isolates (Table S3). Of these, five were located in genes associated with specific functions: histidine metabolism (*hutG*, formiminoglutamase), quorum sensing (*sinR, LysR*-family transcriptional regulator *SinR*), biofilm formation (*rpoS*, RNA polymerase nonessential primary-like sigma factor) and beta-lactam resistance (ompF, outer membrane protein F precursor). The remaining three indels were found in *yhjK* (c-di-GMP phosphodiesterase), a gene encoding an N-acylmannosamine kinase, and a gene for a putative secreted protein.

To explore the possible impact of the observed variation on bacterial function, we determined what the likely coding consequences of 95 SNPs found among the cluster isolates were. This revealed that 17 SNPs were in non-coding regions, 30 SNPs created synonymous changes, 48 SNPs created non-synonymous changes and six were loss-of-function mutations (Table S4).

Of those SNPs overlapping coding regions, these occurred in: hypothetical proteins (*n*=18), metabolism (*n*=12, *pdxA*, *leuA*, *argR*, *metE*, *malZ*, *guaA*, *glpR*, *plsB*, *fabG*, *yiaQ*, *cyoE*, *ubiX*), membrane-bound and/or transport-associated (*n*=4, *kefC*, *potI*, *yjeP*, *yjeM*) and virulence and/or host adaptation (*n*=3, *yehU*, *narZ*, *gtrB*), among others (*n*=10) (Table S4). Of those with a known or probable function, those involved with metabolism were the largest group and included genes involved in amino acid metabolism: *pdxA* (biosynthesis amino acid metabolism coenzyme, pyridoxine 5′-phosphate), *leuA* (leucine synthesis), *argR* (regulator of l-arginine biosynthesis), *metE* (methionine biosynthesis) and *guaA* (GMP biosynthesis, prior to purine synthesis) [46,48], as well as fatty acid metabolism: *glpR* (glycerol-3-phosphate uptake operon repressor), *plsB* (synthesis of LPS fatty acid precursor, CDP-diacylglycerol) and *fabG* (fatty acid chain elongation) [49,51]. The remaining genes were likely involved in carbohydrate utilization (*yiaQ*), maltose utilization (*malZ*), haem O production (cyoE) and ubiquinone biosynthesis (*ubiX*) [52,54].

### AMR and plasmid typing

*In silico* antimicrobial analysis of the case cluster isolates identified a uniform genotypic AMR profile across all isolates sampled (Fig. 2a) except B1 and D3-3 (Fig. 2b, Table S5). These resistant isolates harboured acquired resistance genes *aph(6)-Id*, *bla*TEM-1, *catA-1*, *dfrA-7*, *sul-1* and *sul-2* that were most likely present on an Mutliple Drug Resistant (MDR) composite transposon as previously described in the H58 *S*. Typhi lineage [55]. All isolates also encoded mutations in *gyrA* or *parC* (Table 1). Plasmid typing also identified the presence of an IncQ1 plasmid in all isolates with the exception, again, of B1 and D3-3 (Table S5). None of the isolates screened for AMR had any genotypic markers (acquired genes) conferring resistance to fosfomycin (e.g. isolate D3-1) despite D3-1 being phenotypically resistant (Table 1, Table S2). To explore the genetic diversity among case cluster isolates (1 from each of cases A, B and C and the 13 from case D) (Table 1) in greater detail, another phylogenetic tree was generated to specifically examine the relationships among the 16 case cluster isolates, including the presence of antibiotic resistance genes (Fig. 2b). Isolate B1 and D3-3 exhibited less presence of resistance genes, showing only mutation associated with fluoroquinolone, with B1 having only *gyrA* S83F and D3-3 both *gyrA* S83F and *parC* G78D. However, all other strains exhibited a highly similar pattern of resistance except for the presence of the *parC* G78D mutation. The calculation of the SNP distance between isolates (Fig. 2c) revealed two main clusters, also evidenced on the phylogenetic tree, with a total of 61 SNPs (with respect to the reference) among the 16 isolates, showing that diversity among isolates from case D (range 1–61 pairwise SNPs) was larger than the diversity among cases A, B and C (range 25–32 pairwise SNPs).

## Discussion

This study detected a household outbreak of a genetically diverse population of *S*. Typhi due to a chronic asymptomatic carrier in close contact with the household. Repeated isolation of *S*. Typhi from case D across a duration of 5 months of the study period suggests the case was a carrier for at least 5 months. It is unlikely that the patient was reinfected during that time, as *S*. Typhi is not endemic in the UK, the same 5-SNP cluster was detected across sampling time points and no travel was reported between the initial (24 September 2020) and follow-up isolations (11 November 2020 and 02 February 2021) (Table 1). The patient also disclosed that she had been treated for *S*. Typhi for clinically diagnosed enteric fever on three occasions, once as a child and on two separate occasions as an adult, over 10 years earlier while in Pakistan. Though the exact diagnosis and treatment of these previous infections were not clear, it is possible that she has been a carrier since childhood and may have had multiple infection events.

In our experience, carriers often present with delayed symptoms. *S*. Typhi may be detected as part of enteric testing undertaken when a case develops secondary gastroenteritis, and the detection is an indicator of gut carriage. However, chronic carriers also develop reactivation when there are changes in host immunity due to a variety of reasons. It is difficult to differentiate between the two in this case, as the patient did not attend appointments for specialist assessment.

Although there is a difficulty in reliably reconstructing the evolutionary relationships of isolates within the H58 lineage due to high clonality and few SNPs from which to build a phylogeny [55]. The greater level of genomic discrimination here was able to reveal that case D was likely to be a carrier. Specifically, this is supported by the amount of genetic variation seen within the cluster, as genetic diversity of chronic carriers has been previously shown to be greater than that of acute case isolates, and gall bladder carriage has been found to increase genetic variation [14]. The epidemiological relationships among isolates, encompassing a heterogeneous genetic population in case D, also point towards these being carriage isolates that gave rise to the likely source of infection for cases A, B and C. Gall bladder carriage has been found to increase genetic variation.

Specifically, these data highlight the complexity in interpreting SNP differences in typhoidal cases with contact tracing. Whereas typically the difficulty is the low mutation rate and distinguishing outbreak cases within an endemic population [6,44], in this case, the household outbreak presented with a heterogeneous population. Incorporating SNP differences in a case definition may not be appropriate, and epidemiological context must always be used alongside genetic difference metrics and may require further investigation. Though *S*. Typhi is not endemic within England, there are reports of cases where no travel or contact with recent travellers is reported [6]. This study highlights the underreporting or unknown status of carriers in England that may account for non-travel cases that would be difficult to link genetically with shedding of diverse populations.

The vast amount of variation observed within a single chronic carrier, as demonstrated in our study, indeed contrasts with the relatively small amount of variation observed over large populations and long periods in endemic settings [43]. This observation raises intriguing questions about the dynamics of *S*. Typhi within individual carriers versus in broader endemic populations. The chronic carrier environment might create unique selective pressures and opportunities for diversification within the bacterial population. For instance, prolonged exposure to antibiotics or the host immune system could drive adaptation and diversification. In this case study, the diversity and population structure of the study isolates suggest that case D is the likely source of infection for the other three cases, and the propensity of non-synonymous SNPs suggests that in-host evolution, within this patient, is a significant driver of the variation observed. Most of the genes with SNPs were hypothetical protein genes, also previously identified as more commonly mutated in carrier isolates than acute case isolates [14]. Of those with a known or probable function, those involved with metabolism were the largest group and included genes involved in amino acid metabolism: *pdxA* (biosynthesis amino acid metabolism coenzyme, pyridoxine 5′-phosphate), *leuA* (leucine synthesis), *argR* (regulator of l-arginine biosynthesis), *metE* (methionine biosynthesis) and *guaA* (GMP biosynthesis, prior to purine synthesis) [46,48,56], as well as fatty acid metabolism: *glpR* (glycerol-3-phosphate uptake operon repressor), *plsB* (synthesis of LPS fatty acid precursor, CDP-diacylglycerol) and *fabG* (fatty acid chain elongation) [49,51]. The remaining genes were likely involved in carbohydrate utilization (*yiaQ*), maltose utilization (*malZ*), haem O production (*cyoE*) and ubiquinone biosynthesis (*ubiX*) [52,54].

Among the genes with known or probable functions, a significant portion was associated with metabolism. Specifically, genes involved in amino acid metabolism, such as *pdxA*, *leuA*, *argR*, *metE* and *guaA*, as well as those involved in fatty acid metabolism, including *glpR*, *plsB* and *fabG*, were prominent. Additionally, genes related to carbohydrate and maltose utilization, haem O production and ubiquinone biosynthesis were identified. In the context of in-host evolution, these genes associated with metabolism, particularly those involved in amino acid and fatty acid metabolism, as well as carbohydrate utilization, haem O production and ubiquinone biosynthesis, are significant in exploiting humans as a rich source of nutrients to support survival and replication [57]. These types of metabolic pathways play crucial roles in the adaptation of *Salmonella* Typhi, via mutations, within the host environment, especially considering its ability to persist in the gall bladder and cause long-term carriage [14]. The identification of SNPs within genes related to these pathways suggests potential adaptations occurring within the host, which may facilitate the bacterium’s ability to survive and persist over extended periods. This variation within chronic carriers may contribute to the persistence and transmission of *S*. Typhi in endemic settings, such as through the shedding of diverse bacterial subpopulations.

Furthermore, understanding how these metabolic processes are altered or optimized in response to the host environment sheds light on the mechanisms underlying long-term carriage and occasional shedding of the pathogen. This insight could have implications for developing strategies to prevent or manage chronic carriage cases, thereby reducing the risk of transmission to others. Additionally, investigating the specific genetic changes within these metabolic pathways may provide clues to the evolutionary dynamics driving the persistence and transmission of *S*. Typhi in carrier individuals.

Three identified genes may be involved in virulence or host adaptation: *gtrB* (O-antigen modification), *yehU* (host adaptation) and *narZ* (virulence) [58,60]. While *gtrB* was not specifically identified previously as mutated in carriage isolates, other O-antigen synthesis or modification genes were implicated [9]. Notably, *narZ* knockout *Salmonella* mutants exhibited significantly increased virulence in an oral mouse model, suggesting its involvement in pathogenicity [58]. The gene is required for carbon starvation-inducible thermotolerance and acid tolerance and is expressed within epithelial cells [58].

Differences identified with AMR profiles between isolates are most likely due to the presence/absence of an IncQ1 plasmid. Both isolates found to not encode the IncQ1 rep type B1 (SRR12806902) and D3-3 (SRR13772107) also did not encode a number of AMR determinants compared to IncQ1 carrying isolates (Table 1). This does indicate that these AMR determinants are most likely encoded on this IncQ1 plasmid.

Another interesting observation was that strains isolated from Patient D (D3-1) after treatment with fosfomycin exhibited resistance to this antibiotic but did not harbour the genotypic AMR marker, such as the presence of fosfomycin-modifying enzymes, including the metalloenzymes FosA, FosB and FosX (Table 1, Fig. 2b, Table S2). Neither was there any detection of point mutations in the *murA* genes, also known to cause fosfomycin resistance, as has previously been described [61]. However, it is known that resistance to fosfomycin can arise rapidly *in vitro* through the loss of active transport mechanisms, which are not easily detected genetically, and this is a possible explanation for the development of resistance in this patient [62]. Despite undergoing repeated treatment, patient D continued shedding *S*. Typhi, supporting the hypothesis that this patient was a chronic carrier. Chronic typhoid is associated with the persistence of *S*. Typhi in the gallbladder [11]. Unfortunately, at present, gallbladder removal remains the only effective treatment for chronic typhoid carriage [63].

The observation that patient D harboured a genetically diverse population of *S*. Typhi raises intriguing questions regarding the transmission dynamics within this household cluster. While patients A, B and C were each infected with distinct isolates from patient D’s diverse pool, it remains uncertain whether they were exposed to a genetically pure population or a mixture of strains. Unfortunately, our sequencing approach, which focused on single colonies, limited our ability to discern the full extent of strain diversity within these individuals. ‘Multi-pick’ sampling could have provided valuable insights into the diversity of *S*. Typhi strains acquired by patients A, B and C. Moreover, the timeline of strain diversification within patient D is a point of interest. It is plausible that the observed diversity may have accumulated over decades, reflecting the long-term carriage characteristic of chronic carriers like patient D. However, it is equally plausible that some of the diversity observed could have emerged over the duration of this study due to selective antibiotic/environmental pressure or that multiple infection events with distinct strains occurred in patient D. Thus, while our findings shed light on the genetic diversity within this household cluster, the limitations of our study underscore the need for caution in interpreting the origins and dynamics of strain diversity. Further research incorporating comprehensive sampling strategies and longitudinal studies is warranted to elucidate the complex interplay between long-term carriage, strain diversity and transmission dynamics in *S*. Typhi infections.

Although further investigation is warranted to establish specific links between individual SNPs or genes and carriage, these findings hint at ongoing host adaptation processes within carriage strains. This underscores the need for continued research to elucidate the mechanisms underlying *Salmonella* carriage and its implications for pathogenesis.

## Current guidelines

Current guidelines [64] suggest a pragmatic approach when considering treating chronic *S*. Typhi carriers. Ciprofloxacin and amoxicillin have been used in historical studies [65,68]; however, the vast majority of recent strains are resistant to these antibiotics. The British Infection Association guidelines have recommended azithromycin or fluoroquinolones, if susceptible, for treatment strategies in chronic carriage [64]. Cholecystectomy may be considered, but only at the risk of surgical complications [69,70]. The complex nature of treating chronic *S*. Typhi requires expert infectious disease advice on a case-by-case basis in discussion with the reference laboratory, and cases need regular follow-up to ensure successful clearance.

## Recommendation for the future

The proportion of cases of enteric fever presenting without a travel history to endemic areas in the previous 28 days has been increasing over the past 3 years [6]. Although this approach has its limitations in defining travel-acquired infections, especially for salmonellosis, where intestinal carriage can persist for decades, further work is required to establish pathways of onward transmission, especially from asymptomatic chronic carriers residing in the UK. Detailed case histories, including travel history, previous infections, exposure to antibiotics abroad and in the UK, are essential to understanding the types and rates of carriage, AMR patterns and clinical outcomes and will greatly contribute to the management of this condition as a public health measure to prevent further cases.

Genomics may have a contribution in establishing genes that may play a part in establishing chronicity. Identifying genetic signatures associated with chronic carriage may enable the active identification of carrier-linked cases. However, without consistent routine monitoring of a risk within endemic populations, including asymptomatic patients, it would be difficult to know whether the carriage occurred in the UK or in an endemic setting over a period of years. Our microbiological processes of only testing one colony from a patient at the point of care limit our understanding of in-host variation of microbial population structures over time or exposures to genetically diverse populations at the source of original infection. We recommend further research to develop a cost-effective evidence-based protocol for testing of multiple colonies in selective cases, such as those with long-term carriage, to ensure optimal clearance regimens.

## Conclusion

We describe a rare heterogeneous household outbreak of *S*. Typhi due to chronic carriage and shedding of an asymptomatic carrier and in-host evolution leading to the diversity of subsequent transmission. Although the index and subsequent cases had not travelled to an endemic country, there was epidemiological evidence of the asymptomatic carrier having travelled to Pakistan over the years and having been treated for *S*. Typhi in the past decade and as a child. This indicates that great care is needed when applying genomic criteria with contact tracing and that chronic carriers may account for ‘non-travel’-related cases in England.

## Supplementary material

10.1099/jmm.0.002070 Uncited Supplementary Material 1.

## Abbreviations

AMR: antimicrobial resistance
GBRU: Gastrointestinal Bacteria Reference Unit
MALDI-TOF: matrix-assisted laser desorption ionization time-of-flight
PO: per oral
SNP: single-nucleotide polymorphism
*S.* Typhi: *Salmonella enterica* serotype Typhi
UKHSA: UK Health Security Agency
