# Supplementary material for: Heterogeneous Salmonella typhi transmission within a household: genomic insights from a chronic carrier
Source: J Med Microbiol. 2025 Sep 22;74(9):002070. doi: 10.1099/jmm.0.002070 (PMC12476145; doi:10.1099/jmm.0.002070)
Supplement: Uncited Supplementary Material 1. [file jmm-74-02070-s001.pdf]

Supplementary Table 1. Line list of strains and accession numbers with year, country of travel/location and the genotype used for the contextual phylogeny and functional SNP analysis

SRR Number	Year	Travel	Organism Identified	Genotyphi	Source
SRR10063002	2019	Bangladesh	*Salmonella Typhi*	4.3.1.1	This study (travellers)
SRR10083011	2019	Pakistan	*Salmonella Typhi*	4.3.1.1.P1	This study (travellers)
SRR10096427	2019	Bangladesh	*Salmonella Typhi*	4.3.1.1	This study (travellers)
SRR10120257	2019	N	*Salmonella Typhi*	4.3.1.1	This study (travellers)
SRR10120266	2019	India	*Salmonella Typhi*	4.3.1.1	This study (travellers)
SRR10120309	2019	Zimbabwe	*Salmonella Typhi*	4.3.1.1.EA1	This study (travellers)
SRR10133421	2019	N	*Salmonella Typhi*	4.3.1.1	This study (travellers)
SRR10198869	2019	Pakistan	*Salmonella Typhi*	4.3.1.1	This study (travellers)
SRR10222058	2019	India	*Salmonella Typhi*	4.3.1.2	This study (travellers)
SRR10222067	2019	Kenya	*Salmonella Typhi*	4.3.1.2.EA3	This study (travellers)
SRR10222068	2019	Pakistan	*Salmonella Typhi*	4.3.1.1	This study (travellers)
SRR10222081	2019	Pakistan	*Salmonella Typhi*	4.3.1	This study (travellers)
SRR10229753	2019	Pakistan	*Salmonella Typhi*	4.3.1.1	This study (travellers)
SRR10229824	2019	Pakistan	*Salmonella Typhi*	4.3.1	This study (travellers)
SRR10232197	2019	N	*Salmonella Typhi*	4.3.1	This study (travellers)
SRR10247493	2019	India	*Salmonella Typhi*	4.3.1.2	This study (travellers)
SRR10270617	2019	Pakistan	*Salmonella Typhi*	4.3.1.1	This study (travellers)
SRR10271439	2019	Saudi arabia	*Salmonella Typhi*	4.3.1	This study (travellers)
SRR10313661	2019	Pakistan	*Salmonella Typhi*	4.3.1.1.P1	This study (travellers)
SRR10314900	2019	Zimbabwe	*Salmonella Typhi*	4.3.1.1.EA1	This study (travellers)
SRR10349432	2019	India	*Salmonella Typhi*	4.3.1.2	This study (travellers)
SRR10420550	2019	India	*Salmonella Typhi*	4.3.1.1	This study (travellers)
SRR10420562	2019	India	*Salmonella Typhi*	4.3.1	This study (travellers)
SRR10467022	2019	Pakistan	*Salmonella Typhi*	4.3.1.1.P1	This study (travellers)
SRR4063811	2016	Zimbabwe	*Salmonella Typhi*	4.3.1.1.EA1	This study (travellers)
SRR5194046	2016	India	*Salmonella Typhi*	4.3.1.2	This study (travellers)
SRR5215711	2016	Pakistan	*Salmonella Typhi*	4.3.1	This study (travellers)
SRR5584965	2017	Pakistan	*Salmonella Typhi*	4.3.1.2	This study (travellers)
SRR5585319	2017	Pakistan	*Salmonella Typhi*	4.3.1.1	This study (travellers)
SRR5631945	2017	India	*Salmonella Typhi*	4.3.1.2	This study (travellers)
SRR5632114	2017	N	*Salmonella Typhi*	4.3.1.1	This study (travellers)
SRR5632322	2017	India	*Salmonella Typhi*	4.3.1	This study (travellers)
SRR5632766	2017	Congo	*Salmonella Typhi*	4.3.1.2	This study (travellers)

SRR5633226	2017	India	*Salmonella Typhi*	4.3.1.2	This study (travellers)
SRR5957753	2016	Pakistan	*Salmonella Typhi*	4.3.1	This study (travellers)
SRR5974901	2017	N	*Salmonella Typhi*	4.3.1.1	This study (travellers)
SRR5974916	2016	No travel	*Salmonella Typhi*	4.3.1.2	This study (travellers)
SRR5982990	2016	India	*Salmonella Typhi*	4.3.1.1	This study (travellers)
SRR5989303	2016	India	*Salmonella Typhi*	4.3.1.2	This study (travellers)
SRR5989305	2016	India, Myanmar	*Salmonella Typhi*	4.3.1.1	This study (travellers)
SRR5989306	2016	India	*Salmonella Typhi*	4.3.1.2	This study (travellers)
SRR5990033	2016	No travel	*Salmonella Typhi*	4.3.1.2	This study (travellers)
SRR6181295	2017	Pakistan	*Salmonella Typhi*	4.3.1.1.P1	This study (travellers)
SRR7108940	2018	United Arab Emirates	*Salmonella Typhi*	4.3.1.1	This study (travellers)
SRR7163922	2017	India	*Salmonella Typhi*	4.3.1.2	This study (travellers)
SRR7165016	2017	India	*Salmonella Typhi*	4.3.1.2	This study (travellers)
SRR7165023	2016	India	*Salmonella Typhi*	4.3.1.2	This study (travellers)
SRR7165359	2017	India	*Salmonella Typhi*	4.3.1.2	This study (travellers)
SRR7165360	2017	Pakistan	*Salmonella Typhi*	4.3.1.1	This study (travellers)
SRR7165375	2017	N	*Salmonella Typhi*	4.3.1.1	This study (travellers)
SRR7165378	2016	India	*Salmonella Typhi*	4.3.1.2	This study (travellers)
SRR7165381	2016	Pakistan	*Salmonella Typhi*	4.3.1.1	This study (travellers)
SRR7165388	2016	India	*Salmonella Typhi*	4.3.1.2	This study (travellers)
SRR7165398	2017	India, Thailand	*Salmonella Typhi*	4.3.1.2	This study (travellers)
SRR7165401	2016	Sweden	*Salmonella Typhi*	4.3.1.1	This study (travellers)
SRR7165404	2017	Zimbabwe	*Salmonella Typhi*	4.3.1.1.EA1	This study (travellers)
SRR7165408	2017	Singapore	*Salmonella Typhi*	4.3.1.2	This study (travellers)
SRR7165414	2016	European cruise - multiple destinations	*Salmonella Typhi*	4.3.1.2	This study (travellers)
SRR7165420	2017	Pakistan	*Salmonella Typhi*	4.3.1	This study (travellers)
SRR7165433	2016	India, Myanmar	*Salmonella Typhi*	4.3.1.2	This study (travellers)
SRR7165445	2017	India	*Salmonella Typhi*	4.3.1.2	This study (travellers)
SRR7165453	2016	India	*Salmonella Typhi*	4.3.1.2	This study (travellers)
SRR7165463	2017	Saudi Arabia	*Salmonella Typhi*	4.3.1.1	This study (travellers)
SRR7165472	2016	No travel	*Salmonella Typhi*	4.3.1.1	This study (travellers)
SRR7165482	2016	India	*Salmonella Typhi*	4.3.1.2	This study (travellers)
SRR7165502	2017	India, Thailand	*Salmonella Typhi*	4.3.1.2	This study (travellers)
SRR7165507	2016	Bangladesh	*Salmonella Typhi*	4.3.1.1	This study (travellers)
SRR7165508	2017	Pakistan	*Salmonella Typhi*	4.3.1.1	This study (travellers)
SRR7165510	2018	India	*Salmonella Typhi*	4.3.1.2	This study (travellers)

SRR7165581	2016	Zimbabwe	*Salmonella Typhi*	4.3.1.1.EA1	This study (travellers)
SRR7165606	2016	India	*Salmonella Typhi*	4.3.1.2	This study (travellers)
SRR7165612	2016	No travel	*Salmonella Typhi*	4.3.1.2	This study (travellers)
SRR7165614	2016	No travel	*Salmonella Typhi*	4.3.1	This study (travellers)
SRR7165626	2016	Pakistan	*Salmonella Typhi*	4.3.1	This study (travellers)
SRR7165629	2017	India, Nepal	*Salmonella Typhi*	4.3.1	This study (travellers)
SRR7165734	2016	India	*Salmonella Typhi*	4.3.1.2	This study (travellers)
SRR7165736	2017	Afghanistan, Pakistan	*Salmonella Typhi*	4.3.1.1	This study (travellers)
SRR7165741	2017	No travel	*Salmonella Typhi*	4.3.1.1	This study (travellers)
SRR7165746	2017	Kenya, Tanzania	*Salmonella Typhi*	4.3.1.2.EA3	This study (travellers)
SRR7165787	2016	India, Myanmar	*Salmonella Typhi*	4.3.1.2	This study (travellers)
SRR7166116	2016	Pakistan	*Salmonella Typhi*	4.3.1.2	This study (travellers)
SRR7166117	2017	India	*Salmonella Typhi*	4.3.1.2	This study (travellers)
SRR7275083	2018	Afghanistan	*Salmonella Typhi*	4.3.1.1	This study (travellers)
SRR7277892	2018	India	*Salmonella Typhi*	4.3.1.2	This study (travellers)
SRR7285691	2018	Bangladesh	*Salmonella Typhi*	4.3.1.1	This study (travellers)
SRR7343888	2018	Zimbabwe	*Salmonella Typhi*	4.3.1.1.EA1	This study (travellers)
SRR7351328	2018	Bangladesh	*Salmonella Typhi*	4.3.1.3.Bdq	This study (travellers)
SRR7358965	2018	India	*Salmonella Typhi*	4.3.1	This study (travellers)
SRR7368919	2018	Kenya, Somalia	*Salmonella Typhi*	4.3.1.2.EA3	This study (travellers)
SRR7368927	2018	No travel	*Salmonella Typhi*	4.3.1.1	This study (travellers)
SRR7402073	2018	Greece	*Salmonella Typhi*	4.3.1.2	This study (travellers)
SRR7439522	2018	India	*Salmonella Typhi*	4.3.1.2	This study (travellers)
SRR7457128	2018	Pakistan	*Salmonella Typhi*	4.3.1.1	This study (travellers)
SRR7458004	2018	No travel	*Salmonella Typhi*	4.3.1.1	This study (travellers)
SRR7475302	2018	Zimbabwe	*Salmonella Typhi*	4.3.1.1.EA1	This study (travellers)
SRR7523087	2018	Pakistan	*Salmonella Typhi*	4.3.1.1.P1	This study (travellers)
SRR7842465	2018	Congo	*Salmonella Typhi*	4.3.1.2.EA3	This study (travellers)
SRR7850607	2018	Congo	*Salmonella Typhi*	4.3.1.2.EA3	This study (travellers)
SRR7884514	2018	India	*Salmonella Typhi*	4.3.1.2	This study (travellers)
SRR7997081	2018	N	*Salmonella Typhi*	4.3.1	This study (travellers)
SRR8117049	2018	India	*Salmonella Typhi*	4.3.1.1	This study (travellers)
SRR8137392	2018	Pakistan	*Salmonella Typhi*	4.3.1.2	This study (travellers)
SRR8142772	2018	Pakistan	*Salmonella Typhi*	4.3.1.1	This study (travellers)
SRR8181664	2018	Pakistan	*Salmonella Typhi*	4.3.1.1.P1	This study (travellers)
SRR8239863	2018	Zimbabwe	*Salmonella Typhi*	4.3.1.1.EA1	This study (travellers)
SRR8269055	2018	Nepal	*Salmonella Typhi*	4.3.1	This study (travellers)

SRR8297292	2018	Pakistan	*Salmonella Typhi*	4.3.1.1	This study (travellers)
SRR8325487	2018	India	*Salmonella Typhi*	4.3.1.1	This study (travellers)
SRR8325501	2018	Nepal	*Salmonella Typhi*	4.3.1.2	This study (travellers)
SRR8366139	2018	Pakistan	*Salmonella Typhi*	4.3.1.1	This study (travellers)
SRR8375233	2018	France	*Salmonella Typhi*	4.3.1.1	This study (travellers)
SRR8474060	2019	India	*Salmonella Typhi*	4.3.1.2	This study (travellers)
SRR8489340	2019	Iraq	*Salmonella Typhi*	4.3.1.2	This study (travellers)
SRR8490672	2018	India	*Salmonella Typhi*	4.3.1.2	This study (travellers)
SRR8490821	2018	Pakistan	*Salmonella Typhi*	4.3.1.2	This study (travellers)
SRR8492464	2018	Bangladesh	*Salmonella Typhi*	4.3.1.1	This study (travellers)
SRR8498996	2018	India	*Salmonella Typhi*	4.3.1.2	This study (travellers)
SRR8499364	2018	Cambodia, Sri Lanka	*Salmonella Typhi*	4.3.1.1	This study (travellers)
SRR8503967	2018	United Arab Emirates	*Salmonella Typhi*	4.3.1.1	This study (travellers)
SRR8508953	2019	Pakistan	*Salmonella Typhi*	4.3.1.2	This study (travellers)
SRR8509417	2019	India	*Salmonella Typhi*	4.3.1.2	This study (travellers)
SRR8514765	2018	India	*Salmonella Typhi*	4.3.1.2	This study (travellers)
SRR8554071	2019	Iraq	*Salmonella Typhi*	4.3.1.2	This study (travellers)
SRR8568760	2017	India, Nepal	*Salmonella Typhi*	4.3.1.2	This study (travellers)
SRR8585094	2019	Pakistan	*Salmonella Typhi*	4.3.1.1	This study (travellers)
SRR8638517	2019	India	*Salmonella Typhi*	4.3.1.2	This study (travellers)
SRR8655495	2019	India	*Salmonella Typhi*	4.3.1	This study (travellers)
SRR8691522	2019	India	*Salmonella Typhi*	4.3.1.1	This study (travellers)
SRR8704729	2019	Pakistan	*Salmonella Typhi*	4.3.1.2	This study (travellers)
SRR8748716	2019	India	*Salmonella Typhi*	4.3.1	This study (travellers)
SRR8775496	2019	Pakistan	*Salmonella Typhi*	4.3.1.1	This study (travellers)
SRR8784398	2019	N	*Salmonella Typhi*	4.3.1	This study (travellers)
SRR8873670	2019	India	*Salmonella Typhi*	4.3.1.2	This study (travellers)
SRR8874211	2019	Uganda	*Salmonella Typhi*	4.3.1.1.EA1	This study (travellers)
SRR8943110	2019	Pakistan	*Salmonella Typhi*	4.3.1.1.P1	This study (travellers)
SRR9261293	2019	Indonesia	*Salmonella Typhi*	4.3.1.2	This study (travellers)
SRR9261342	2019	India	*Salmonella Typhi*	4.3.1.2	This study (travellers)
SRR9261695	2019	Pakistan	*Salmonella Typhi*	4.3.1	This study (travellers)
SRR9273941	2019	India	*Salmonella Typhi*	4.3.1.2	This study (travellers)
SRR9273977	2019	Bangladesh	*Salmonella Typhi*	4.3.1.1	This study (travellers)
SRR9274370	2019	India	*Salmonella Typhi*	4.3.1.1	This study (travellers)
SRR9287407	2019	Pakistan	*Salmonella Typhi*	4.3.1.1	This study (travellers)
SRR9304279	2019	Zimbabwe	*Salmonella Typhi*	4.3.1.1.EA1	This study (travellers)

SRR9335596	2019	Zimbabwe	*Salmonella Typhi*	4.3.1.1.EA1	This study (travellers)
SRR9617149	2019	Pakistan	*Salmonella Typhi*	4.3.1.1.P1	This study (travellers)
SRR9625950	2019	Saudi arabia	*Salmonella Typhi*	4.3.1	This study (travellers)
SRR9649500	2019	India	*Salmonella Typhi*	4.3.1	This study (travellers)
SRR9719753	2019	Bangladesh	*Salmonella Typhi*	4.3.1.1	This study (travellers)
SRR9923798	2019	Pakistan	*Salmonella Typhi*	4.3.1.1.P1	This study (travellers)
SRR9944955	2019	China	*Salmonella Typhi*	4.3.1.2	This study (travellers)
ERR1017048	2006	Unknown	*Salmonella Typhi*	3.0.1	This study (travellers)
ERR213233	2011	Fiji	*Salmonella Typhi*	4.2.1	Wong et al, 2015 (global framework)
ERR213272	1995	Fiji	*Salmonella Typhi*	4.2.3	Wong et al, 2015 (global framework)
ERR279098	2011	Malawi	*Salmonella Typhi*	2.2.0	Wong et al, 2015 (global framework)
ERR326624	2012	Indonesia	*Salmonella Typhi*	2.1.9	Wong et al, 2015 (global framework)
ERR326672	2010	Laos	*Salmonella Typhi*	2.2.3	Wong et al, 2015 (global framework)
ERR331206	2012	Cambodia	*Salmonella Typhi*	4.3.1	Wong et al, 2015 (global framework)
ERR331225	2011	Indonesia	*Salmonella Typhi*	2.1.3	Wong et al, 2015 (global framework)
ERR331236	2011	Indonesia	*Salmonella Typhi*	4.1.0	Wong et al, 2015 (global framework)
ERR331237	2011	Indonesia	*Salmonella Typhi*	3.0.0	Wong et al, 2015 (global framework)
ERR331264	2012	Fiji	*Salmonella Typhi*	4.2.1	Wong et al, 2015 (global framework)
ERR331265	2012	Fiji	*Salmonella Typhi*	4.2.2	Wong et al, 2015 (global framework)
ERR331292	2011	Bangladesh	*Salmonella Typhi*	4.3.1	Wong et al, 2015 (global framework)
ERR331313	2010	Laos	*Salmonella Typhi*	3.5.2	Wong et al, 2015 (global framework)
ERR331329	2010	Laos	*Salmonella Typhi*	3.4.0	Wong et al, 2015 (global framework)
ERR331332	2010	Laos	*Salmonella Typhi*	3.2.1	Wong et al, 2015 (global framework)
ERR337992	2012	Bangladesh	*Salmonella Typhi*	3.3.0	Wong et al, 2015 (global framework)
ERR338008	2007	South Africa	*Salmonella Typhi*	1.1.2	Wong et al, 2015 (global framework)
ERR338025	2010	South Africa	*Salmonella Typhi*	2.4.0	Wong et al, 2015 (global framework)
ERR338027	2010	South Africa	*Salmonella Typhi*	3.3.1	Wong et al, 2015 (global framework)
ERR338032	2011	South Africa	*Salmonella Typhi*	2.5.0	Wong et al, 2015 (global framework)
ERR338041	2012	South Africa	*Salmonella Typhi*	2.4.1	Wong et al, 2015 (global framework)
ERR338049	2012	South Africa	*Salmonella Typhi*	3.1.0	Wong et al, 2015 (global framework)
ERR338051	2012	Indonesia	*Salmonella Typhi*	3.1.2	Wong et al, 2015 (global framework)
ERR338061	2011	Indonesia	*Salmonella Typhi*	2.1.8	Wong et al, 2015 (global framework)
ERR338079	2012	Samoa	*Salmonella Typhi*	3.5.4	Wong et al, 2015 (global framework)
ERR338151	2008	Kenya	*Salmonella Typhi*	4.3.1	Wong et al, 2015 (global framework)
ERR343250	2002	Western Asia	*Salmonella Typhi*	2.1.4	Wong et al, 2015 (global framework)
ERR343257	2003	Tonga	*Salmonella Typhi*	4.2.0	Wong et al, 2015 (global framework)
ERR343301	2010	India	*Salmonella Typhi*	2.0.1	Wong et al, 2015 (global framework)

ERR343305	2010	Samoa	*Salmonella Typhi*	3.5.3	Wong et al, 2015 (global framework)
ERR343308	2010	South America	*Salmonella Typhi*	2.3.2	Wong et al, 2015 (global framework)
ERR343316	2011	Samoa	*Salmonella Typhi*	3.5.4	Wong et al, 2015 (global framework)
ERR343317	2011	Nepal	*Salmonella Typhi*	2.2.0	Wong et al, 2015 (global framework)
ERR343321	2011	Mexico	*Salmonella Typhi*	2.0.2	Wong et al, 2015 (global framework)
ERR343325	2012	India	*Salmonella Typhi*	2.2.4	Wong et al, 2015 (global framework)
ERR349339	2011	Nepal	*Salmonella Typhi*	4.3.1	Wong et al, 2015 (global framework)
ERR352262	2011	Tonga	*Salmonella Typhi*	2.3.5	Wong et al, 2015 (global framework)
ERR352269	2011	China	*Salmonella Typhi*	2.3.4	Wong et al, 2015 (global framework)
ERR352285	2011	India	*Salmonella Typhi*	3.2.1	Wong et al, 2015 (global framework)
ERR352288	2010	Papua New Guinea	*Salmonella Typhi*	2.1.7	Wong et al, 2015 (global framework)
ERR352303	2010	Bangladesh	*Salmonella Typhi*	3.2.2	Wong et al, 2015 (global framework)
ERR352337	2011	Unknown	*Salmonella Typhi*	4.1.0	Wong et al, 2015 (global framework)
ERR352453	2011	Unknown	*Salmonella Typhi*	1.1.4	Wong et al, 2015 (global framework)
ERR352461	2010	Bangladesh	*Salmonella Typhi*	2.0.0	Wong et al, 2015 (global framework)
ERR352467	2011	Indonesia	*Salmonella Typhi*	2.1.5	Wong et al, 2015 (global framework)
ERR352472	2011	Indonesia	*Salmonella Typhi*	2.1.0	Wong et al, 2015 (global framework)
ERR352487	2010	Papua New Guinea	*Salmonella Typhi*	2.1.7	Wong et al, 2015 (global framework)
ERR352602	2011	DRC	*Salmonella Typhi*	2.5.1	Wong et al, 2015 (global framework)
ERR353338	1999	Argentina	*Salmonella Typhi*	2.0.0	Wong et al, 2015 (global framework)
ERR357441	2012	Myanmar	*Salmonella Typhi*	3.3.1	Wong et al, 2015 (global framework)
ERR357443	2012	India	*Salmonella Typhi*	3.0.2	Wong et al, 2015 (global framework)
ERR357631	2012	Samoa	*Salmonella Typhi*	2.2.1	Wong et al, 2015 (global framework)
ERR357652	1993	Vietnam	*Salmonella Typhi*	1.2.1	Wong et al, 2015 (global framework)
ERR357767	2011	India	*Salmonella Typhi*	2.2.2	Wong et al, 2015 (global framework)
ERR357775	2011	Indonesia	*Salmonella Typhi*	0.0.2	Wong et al, 2015 (global framework)
ERR357797	2011	India	*Salmonella Typhi*	4.3.1	Wong et al, 2015 (global framework)
ERR357831	2012	Indonesia	*Salmonella Typhi*	2.1.6	Wong et al, 2015 (global framework)
ERR360456	2003	Pakistan	*Salmonella Typhi*	2.0.1	Wong et al, 2015 (global framework)
ERR360484	2009	Algeria	*Salmonella Typhi*	0.1.0	Wong et al, 2015 (global framework)
ERR360486	1998	Peru	*Salmonella Typhi*	0.1.3	Wong et al, 2015 (global framework)
ERR360505	2001	Cameroon	*Salmonella Typhi*	0.1.1	Wong et al, 2015 (global framework)
ERR360511	1976	DRC	*Salmonella Typhi*	1.1.3	Wong et al, 2015 (global framework)
ERR360627	2005	Algeria	*Salmonella Typhi*	1.1.1	Wong et al, 2015 (global framework)
ERR360628	2007	Cameroon	*Salmonella Typhi*	2.3.1	Wong et al, 2015 (global framework)
ERR360645	2009	France (African meal)	*Salmonella Typhi*	3.1.1	Wong et al, 2015 (global framework)
ERR360655	2000	Cameroon	*Salmonella Typhi*	0.0.1	Wong et al, 2015 (global framework)

ERR360668	1999	Algeria	*Salmonella Typhi*	0.1.2	Wong et al, 2015 (global framework)
ERR360696	2000	Indonesia	*Salmonella Typhi*	3.5.0	Wong et al, 2015 (global framework)
ERR360747	2010	Cambodia	*Salmonella Typhi*	3.4.0	Wong et al, 2015 (global framework)
ERR360803	2012	Malawi	*Salmonella Typhi*	4.1.1	Wong et al, 2015 (global framework)
ERR422811	2011	Bangladesh	*Salmonella Typhi*	2.3.3	This study (travellers)
ERR422822	2011	Bangladesh	*Salmonella Typhi*	3.3.0	This study (travellers)

Supplementary Table 2. Phenotypic data (MIC) and genotype data of antimicrobial resistance testing.

	Patient A (A1) S RR12672341			Patient B (B1) SRR12806902			Patient C (C1) S RR12743431			Patient D (D1-1) SRR12807764			Patient D (D2-1) SRR131163225			Patient D (D3-1) SRR13833611		
Antibiotics	MIC	Antibiotic Susceptibility Interpretation	Resistance Gene	MIC	Antibiotic Susceptibility Interpretation	Resistance Gene	MIC	Antibiotic Susceptibility Interpretation	Resistance Gene	MIC	Antibiotic Susceptibility Interpretation	Resistance Gene	MIC	Antibiotic Susceptibility Interpretation	Resistance Gene	MIC	Antibiotic Susceptibility Interpretation	Resistance Gene
Amikacin	<=4	R		<=4	R		<=4	R		<=4	R		<=4	R		<=4	R	
Amox-clav	8/2	S		<=2/2	S	-	8/2	S		8/2	S		8/2	S		8/2	S	
Ampicilin	>8	R	TEM-1	<=2/2	S	-	>8	R	TEM-1	>8	R	TEM-1	>8	R	TEM-1	>8	R	TEM-1
Aztreonam	<=1	-		<=1	-		<=1			<=1			<=1			<=1		
Cefotaxime	<=0.5	S			S		<=0.5	S		<=0.5	S		<=0.5	S		<=0.5	S	
Ceftazidime	<=0.5	S			S		1	S		<=0.5	S		<=0.5	S		<=0.5	S	
Cephalexin	4	R	TEM-1	4	R	TEM-1	8	R	TEM-1	4	R	TEM-1	4	R	TEM-1	4	R	TEM-1
Ciprofloxacin	1	R	gyrA C_ [83:S-F]; parC_ [78:G-D]	1	R	gyrA C_ [83:S-F]	1	R	gyrA C_ [83:S-F]	1	R	gyrA C_ [83:S-F]	1	R	gyrA C_ [83:S-F]	1	R	gyrA C_ [83:S-F]
Colistin	<=1	-		<=1			<=1			<=1			<=1			<=1		
Ertapenem	<=0.25	S	-	<=0.25	S	-		S	-	<=0.25	S	-	<=0.25	S	-	<=0.25	S	-
Fosfomycin	<=16	S		<=16	S		<=0.25	S		<=16	S		<=16	S		>64	[1]R	
Gentamicin	<=1	R	aph(6)-Id,strB;aac(6)-Iy];strA	<=1	R	aac(6)-Iy	<=1	R	aph(6)-Id,strB;aac(6)-Iy[u];strA	<=1	R	aac(6)-Iy;aph(6)-Id,strB;strA	<=1	R	aac(6)-Iy;aph(6)-Id,strB;strA	<=1	R	aac(6)-Iy;aph(6)-Id,strB;strA
Imipenem	<=1	S	-	<=1	S	-	<=1	S	-	<=1	S	-	<=1	S	-	<=1	S	-
Meropenem	<=1	S	-	<=1	S	-	<=1	S	-	<=1	S	-	<=1	S	-	<=1	S	-
Nitrofurantoin	32	S		32	S		32	S		32	S		32	S		32		
Piperacillin-tazobactam	<=4/4	S		<=4/4	S		<=4/4	S		<=4/4	S		<=4/4	S		<=4/4		
Trimethoprim	>4	R	dfrA-7	<=1	S	-	>4	R	dfrA-7	>4	R	dfrA-7	>4	R	dfrA-7	>4	R	dfrA-7
Trimethoprim-sulfamethoxazole	>4/76	S	sul-1;sul-2	<=1/19	S	sul-1;sul-2	>4/76	R	sul-1;sul-2	>4/76	R	sul-1;sul-2	>4/76	R	sul-1;sul-2	>4/76	R	sul-1;sul-2

[1]Fosfomycin – R, [1] Post Fosfomycin use as eradication therapy. No specific gene detected and may be due to other mechanisms such as efflux pumps.

Supplementary Table 3. Indel analysis

Genome position (Ty2)	Base	Change (coding sequence)	Change (protein)	Gene
1953882	AT	117delA	K39fs	STY1043
1980190	T	504_505dupGG	D169fs	STY1002 (ompF)
2162522	ACGCGCGCCGMTG	899_910delCAMCGGCGCGCG	p.A300_R303del	STY0821 (hutG)
2631888	AT	543delA	K181fs	STY0341 (sinR)
2900995	GC	933delG	P312fs	STY3049 (rpoS)
3345563	TG	232delC	H78fs	STY3516
4036236	CT	1863delT	G622fs	STY4188 (yhjK)

Indel analysis including genome position, base change, gene affected and function. Only indels predicted to have an impact on gene function by SnpEff are shown. Identical mutations were found in all 16 study isolates.

Supplementary Table 4. Mutation analysis including genome position, base change, gene affected and function

Genome position (Ty2)	Base	Change	Gene	Function
32912	T	SYNONYMOUS	STY0035	putative membrane protein
45349	T	Q348*	STY0046	possible sulfatase
77927	C	SYNONYMOUS	STY0077 (carB)	carbamoyl-phosphate synthase large chain
99807	T	R409*	STY0101 (kefC)	glutathione-regulated potassium-efflux system protein KefC
103749	T	A173T	STY0106 (pdxA)	pyridoxal phosphate biosynthetic protein PdxA
133396	C	SYNONYMOUS	STY0131 (leuB)	3-isopropylmalate dehydrogenase
134635	G	D170A	STY0132 (leuA)	2-isopropylmalate synthase
137615	A	SYNONYMOUS	STY0135 (ilvI)	acetolactate synthase isozyme III large subunit
209543	A	SYNONYMOUS	STY0200 (panB)	3-methyl-2-oxobutanoate hydroxymethyltransferase
331095	T	SYNONYMOUS	STY2812 (purL)	phosphoribosylformylglycineamide synthetase
335417	A	G23S	STY2809 (yfhA)	putative transcriptional regulator
405740	T	SYNONYMOUS	STY2756 (ratC)	conserved hypothetical protein
416602	T	Q334H	STY2751 (guaA)	GMP synthase
452613	A	SYNONYMOUS	STY2718 (narQ)	nitrate/nitrite sensor protein
576151	A	SYNONYMOUS	STY2591	putative transcriptional regulator
582662	A	R41Q	STY2586 (ubiX)	putative decarboxylase
636888	A	SYNONYMOUS	STY2531 (pqaB)	melittin resistance protein
743312	A	SYNONYMOUS	STY2431	putative hydrolase
772207	T	NON-CODING		
783821	A	G538S	STY2389 (yehU)	putative two-component system sensor kinase
917827	A	SYNONYMOUS	STY2258 (pduS)	putative ferredoxin
924301	G	L60P	STY2249 (pduH)	PduH protein
962377	T	NON-CODING		
991507	T	SYNONYMOUS	STY2194	putative membrane protein

1113523	C	T52A	STY1980	conserved hypothetical protein
1132089	T	H208Y	STY1958	putative membrane protein
1158993	T	G243D	STY1929	putative Na+/H+ exchanger
1172258	T	G444S	STY1914	hydrogenase-1 large chain
1186170	T	SYNONYMOUS	STY1901 (prfA)	peptide chain release factor 1
1265734	T	R741H	STY1793 (katE)	catalase HPII
1387715	T	G18S	STY1665	putative NADH reducing dehydrogenase
1387915	T	SYNONYMOUS	STY1664	putative ferredoxin-like protein, cytoplasmic membrane
1394264	T	R146H	STY1656	conserved hypothetical protein
1452382	T	S2N	STY1579	putative membrane transport protein
1461181	T	P47Q	STY1569	conserved hypothetical protein
1521824	C	I405M	STY1503	putative hydrolase
1537875	A	R268H	STY1488 (narZ)	respiratory nitrate reductase 2 alpha chain
1594389	A	W9*	STY1434	putative membrane protein
1606644	A	NON-CODING		
1628518	T	NON-CODING		
1706354	T	SYNONYMOUS	STY1315 (yciA)	putative acyl-coA hydrolase
1792100	A	A64V	STY1234 (fabG)	3-oxoacyl-[acyl-carrier protein] reductase
1813653	T	R29C	STY1211 (flgM)	negative regulator of flagellin synthesis
1847931	T	G385E	STY1169	putative transporter
1848737	A	SYNONYMOUS	STY1169	putative transporter
1929458	T	NON-CODING		
2031759	A	SYNONYMOUS	STY0958 (ftsK)	cell division protein FtsK
2074622	G	L122P	STY0913 (potI)	putrescine transport system permease protein PotI
2360739	A	SYNONYMOUS	STY0619 (apeE)	outer membrane esterase
2372373	T	R198C	STY0606 (gtrB)	bactoprenol glucosyltransferase
2411973	C	NON-CODING		
2413083	C	NON-CODING		
2417992	G	F55S	STY0556	outer membrane protein
2495258	T	R184C	STY0481 (cyoE)	cytochrome o ubiquinol oxidase C subunit
2531510	T	W401*	STY0439 (malZ)	maltodextrin glucosidase
2550511	A	Q325*	STY0418 (adrA)	adrA protein
2652891	T	M19I	STY0318	hypothetical protein
2782592	A	NON-CODING		
2894508	A	P209L	STY3040 (hyi)	putative hydroxypyruvate isomerase

3026219	A	W284*	STY3167	probable amino acid transport protein
3122838	A	NON-CODING		
3145068	A	SYNONYMOUS	STY3310 (gsp)	glutathionylspermidine synthetase/amidase
3181971	T	G78D	STY3351 (parC)	topoisomerase IV subunit A
3187117	A	L579F	STY3359 (parE)	topoisomerase IV subunit B
3242075	A	G193S	STY3413	conserved hypothetical protein
3319870	A	NON-CODING		
3367583	G	E155G	STY3540 (argR)	arginine repressor
3433273	T	A706D	STY3594 (metE)	5-methyltetrahydropteroyltriglutamate- homocysteine methyltransferase
3491875	G	NON-CODING		
3504985	T	L41F	STY3665	hypothetical protein
3556040	T	SYNONYMOUS	STY3721 (thiC)	thiamine biosynthesis protein
3655640	G	NON-CODING		
3687910	T	SYNONYMOUS	STY3843 (fdhE)	FdhE protein
3762096	A	NON-CODING		
3810476	A	G267S	STY3955 (torC)	hypothetical protein
3970926	C	T71A	STY4121 (yiaQ)	putative hexulose-6-phosphate synthase
4017293	T	L262F	STY4172 (dppF)	dipeptide transport ATP-binding protein DppF
4134945	T	G54C	STY4280 (glpR)	glycerol-3-phosphate regulon repressor
4135486	T	P234L	STY4280 (glpR)	glycerol-3-phosphate regulon repressor
4150617	A	SYNONYMOUS	STY4290 (feoB)	ferrous iron transport protein B
4203405	T	SYNONYMOUS	STY4339 (yheS)	probable ABC transporter ATP-binding protein
4213080	T	SYNONYMOUS	STY4352 (fusA)	elongation factor G
4286819	T	A519T	STY4431 (plsB)	glycerol-3-phosphate acyltransferase
4383061	T	NON-CODING		
4526001	C	NON-CODING		
4547374	A	G228R	STY4705 (yjeM)	putative amino acid permease
4549789	A	P752L	STY4707 (yjeP)	putative membrane protein
4552284	A	SYNONYMOUS	STY4708 (psd)	phosphatidylserine decarboxylase proenzyme
4578756	A	G233R	STY4733 (aidB)	probable acyl Co-A dehydrogenase
4583687	T	SYNONYMOUS	STY4739 (sgaT)	putative transport protein SgaT
4653479	A	SYNONYMOUS	STY4811	putative exported protein
4665579	A	NON-CODING		
4686828	A	NON-CODING		

4695636	A	SYNONYMOUS	STY4850	DEAD-box helicase-related protein
4753255	T	SYNONYMOUS	STY4909	conserved hypothetical protein

Supplementary Table 5. Table showing summary of PlasmidFinder 2.0 results of S. Typhi isolates from the cases and chronic carrier. Reference No. is based on the patient (A-D) and the individual colony number (-X) that was isolated from the original clinical sample and sequenced. All isolates expect B1 and D3-3 were found to carry an IncQ1 plasmid.

Patient	Reference No.	Case/ Carrier	Sex	Age	SRA	Sample Date	Plasmid Replicon	Percent Identity	Query / Template length	Contig Name	Length (bp)	Position in contig (bp)	Accession number
A	A1	Case	F	20	SRR12672341	05/09/2020	IncQ1	100	524 / 796	46	length=4386	3863..4386	M28829
B	B1	Case	F	15	SRR12806902	23/09/2020	N/A	N/A	N/A	N/A	N/A	N/A	N/A
C	C1	Case	M	11	SRR12743431	11/09/2020	IncQ1	100	524 / 796	53	length=4386	1..524	M28829
D	D1-1	Carrier	F	78	SRR12807764	24/09/2020	IncQ1	100	524 / 796	43	length=4386	1..524	M28829
D	D2-1	Carrier	F	78	SRR13163225	11/11/2020	IncQ1	100	524 / 796	45	length=4386	3863..4386	M28829
D	D3-1	Carrier	F	78	SRR13833611	02/02/2021	IncQ1	100	524 / 796	47	length=4386	1..524	M28829
D	D3-2	Carrier	F	78	SRR15334932	02/02/2021	IncQ1	100	524 / 796	56	length=4386	3863..4386	M28829
D	D3-3	Carrier	F	78	SRR13772107	02/02/2021	N/A	N/A	N/A	N/A	N/A	N/A	N/A
D	D3-4	Carrier	F	78	SRR13833659	02/02/2021	IncQ1	100	524 / 796	51	length=4386	3863..4386	M28829
D	D3-5	Carrier	F	78	SRR15334714	02/02/2021	IncQ1	100	529 / 796	46	length=5285	71..599	M28829
D	D3-6	Carrier	F	78	SRR15334737	02/02/2021	IncQ1	100	524 / 796	60	length=4386	3863..4386	M28829
D	D3-7	Carrier	F	78	SRR15334694	02/02/2021	IncQ1	100	524 / 796	59	length=4386	1..524	M28829
D	D3-8	Carrier	F	78	SRR13833654	02/02/2021	IncQ1	100	524 / 796	56	length=4386	3863..4386	M28829
D	D3-9	Carrier	F	78	SRR15340843	02/02/2021	IncQ1	100	524 / 796	50	length=4386	3863..4386	M28829
D	D3-10	Carrier	F	78	SRR15334775	02/02/2021	IncQ1	100	524 / 796	54	length=4386	1..524	M28829
D	D3-11	Carrier	F	78	SRR15334815	02/02/2021	IncQ1	100	524 / 796	54	length=4386	3863..4386	M28829
